# Bibliometric analysis of research on Alzheimer’s disease and non-coding RNAs: Opportunities and challenges

**DOI:** 10.3389/fnagi.2022.1037068

**Published:** 2022-10-18

**Authors:** Xinxing Fei, Shiqi Wang, Jiyang Li, Qiu Zeng, Yaqian Gao, Yue Hu

**Affiliations:** ^1^Department of Psychiatry, Chengdu Eighth People’s Hospital (Geriatric Hospital of Chengdu Medical College), Chengdu, China; ^2^Rehabilitation Medicine Center and Institute of Rehabilitation Medicine, West China Hospital, Sichuan University, Chengdu, China; ^3^Key Laboratory of Rehabilitation Medicine in Sichuan Province, Chengdu, China; ^4^Department of Rehabilitation Medicine, The Affiliated Hospital of Southwest Medical University, Luzhou, China; ^5^Department of Rehabilitation Medicine, The First Affiliated Hospital of Chengdu Medical College, Chengdu, China

**Keywords:** Alzheimer’s disease, ncRNAs, bibliometrics, research trends, hotspots, frontiers, neurodegenerative diseases

## Abstract

**Background:**

Non-coding RNAs (ncRNA) are a kind of RNA that does not encode protein, which play an important role in Alzheimer’s disease (AD). However, there is a lack of bibliometric analysis and visualization analysis of the research related to AD and ncRNAs.

**Materials and methods:**

Literature related to AD and ncRNAs in the last decade were searched through the Web of Science Core Collection (WOSCC). The relevant information from all the searched articles was collected. The bibliometric visualization website, CiteSpace, and VOSviewer were used for visualization analysis of countries/regions, institutions, authors, and keywords.

**Results:**

In total, 1,613 kinds of literature were published in the field. Literature in this field were published in 494 journals. The *Journal of Alzheimer’s Disease* was the most popular journal. China, Louisiana State University System, and Lukiw WJ were the countries/regions, institutions, and authors with the highest scientific productivity, respectively. The research hotspots in this field focused on the role and mechanism of ncRNAs, especially microRNAs, in AD. The level of research was mainly based on basic research, focusing on animal and cellular levels, and related to proteomics. “Circular RNAs,” “regulation of neuroinflammation,” and “tau protein” were the future research directions.

**Conclusion:**

Taken together, the field of AD and ncRNAs is developing well. The research hotspots and frontiers in this field can provide a reference for researchers to choose their research direction.

## Introduction

Alzheimer’s disease (AD) is a neurodegenerative disease with insidious onset, characterized by progressive cognitive dysfunction and memory impairment (Morató et al., 2022). The main pathological features of AD are decreased a number of cortical neurons, nerve fiber tangles, and senile plaque deposition (Counil and Krantic, 2020; Tran et al., 2022). With the increase of age, the incidence of AD increases gradually, which seriously affects the quality of life and brings a great burden to families. At present, AD has become an important public health problem (Clark et al., 2022; Wang Q. et al., 2022).

Non-coding RNAs (ncRNAs) are a kind of RNA that does not encode protein, which play an important role in tumor, cardiovascular and nervous system diseases (Wang S. et al., 2022). For example, it has been reported that nuclear paraspeckle assembly transcript 1 (NEAT1) lncRNA, an ncRNA, can be used as a potential blood-based biomarker for AD (Khodayi et al., 2022). Indeed, the role of ncRNAs in nervous system diseases has been further refined, and in-depth studies on the physiological functions of a specific ncRNA have gradually shown a potential correlation with AD (Jiang et al., 2022). For example, miR-143-3p has been found to inhibit abnormal Tau phosphorylation in AD and amyloid processing in amyloid precursor protein (Wang L. et al., 2022).

Bibliometrics can analyze the research trend in a particular field in a certain period time (Wang et al., 2020). Through the quantitative and qualitative analysis of the literature in the field *via* mathematical and statistical methods, the research hotspots and research frontiers can be revealed (Fei et al., 2022; Zhang Z. et al., 2022). Moreover, bibliometrics has played a crucial role in neurology, psychiatry, cardiovascular disease, and many other disciplines. Besides, data visualization tools such as CiteSpace and VOSviewer have also been widely used in bibliometric analysis (Zhang Y. et al., 2022).

With the deepening of research, new ncRNAs have been continuously isolated and identified, and increased research have been conducted to explore the relationship between AD and ncRNAs in the past 10 years. However, up to now, there is a lack of bibliometric analysis and visualization analysis of the research related to AD and ncRNAs. Thus, our study searches the literature related to AD and ncRNAs in the last decade through the Web of Science Core Collection (WOSCC), aiming to reveal the research trends, research hotspots, and frontiers in this field *via* the means of bibliometric analysis.

## Materials and methods

### Search strategy

**FIGURE 1:**
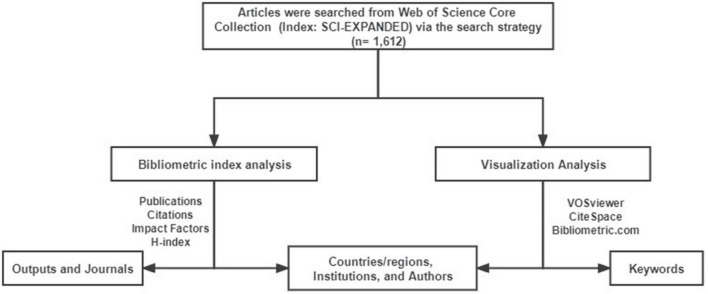
Flow chart of literature research, bibliometric analysis, and visualization analysis.

The specific search strategy is shown in Table 1. The search was completed independently by two researchers on August 21, 2022, and then bibliometric analysis and visual analysis were carried out after the search results were consistent. The specific process is shown in Figure 1.

**TABLE 1:** Search strategy.

Item	Search strategy
**#1**	((((((((TS = (”non-coding RNA*”)) OR TS = (”non-coding RNA*”)) OR TS = (miRNA*)) OR TS = (microRNA*)) OR TS = (lncRNA*)) OR TS = (”long non-coding RNA*”)) OR TS = (”long non-coding RNA*”)) OR TS = (circRNA*)) OR TS = (”circular RNA*”)
**#2**	((((TS = (”Alzheimer’s disease*”)) OR TS = (”Senile Dementia”)) OR TS = (”Alzheimer’s Disease*”)) OR TS = (”Alzheimer Type Dementia”)) OR TS = (”Alzheimer Syndrome*”)
**#3**	**#1** AND **#2**

Search in: Web of Science Core Collection; Editions: Science Citation Index Expanded (SCI-EXPANDED)—1900-present; Date range: 2012-01-01 to 2021-12-31.

*Represents any group of characters, including no character.

### Data collection and bibliometric index statistics

The relevant information of all the searched articles was exported, and the bibliometric indicators were counted by Excel, including the annual number of publications, citation frequency, average citation frequency, journal name, journal impact factor (IF), SCImago Journal Rank (SJR) Indicator, publication country/region, publication institution, author, and author’s H-index.

### Visualization analysis

The map of countries/regions cooperation was made by the bibliometric visualization website^1^. The maps of institutions’ cooperation and authors’ cooperation were made by VOSviewer (Version 1.6.16). Considering that research hotspots and frontiers are an important part of the bibliometric analysis in a professional field, it is of great significance to summarize and discuss the research hotspots in this field. Therefore, when analyzing keywords, we used CiteSpace (Version V5.8.R3) software to conduct co-occurrence, clustering, and burst analysis of keywords. The original data of all search results used for visualization analysis have been uploaded to Supplementary Data Sheet 1. And the parameter settings of each visualization tool have been uploaded to Supplementary Data Sheet 2.

All the above steps were completed by two researchers independently. When the results of data processing were inconsistent, a third researcher intervened and a unified result was obtained after discussion. The content of the discussion included whether the search strategy (punctuation, date range, database selection, etc.) and the format of the exported data were correct, as well as whether the parameters selection of visualization tools was consistent.

## Results

### Outputs and journals

**FIGURE 2:**
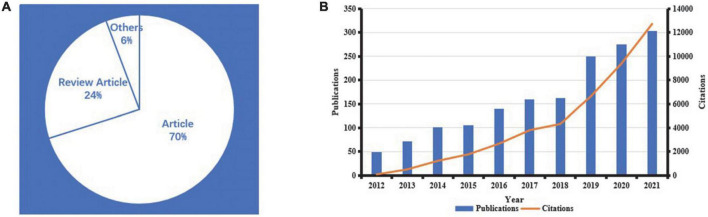
Document type distribution **(A)** and publications/citations situation **(B)** in the field of AD and ncRNAs in the past decade.

Totally, 1,613 kinds of literature were published in the field of AD and ncRNAs in the last decade, among which article and review accounted for 70 and 24% of all document types, respectively (Figure 2A). By the end of the search date (August 28, 2022), the total citations reached 50,937, with an average of 31.6 citations per literature.

We summarized the annual publications and citations of literature in this field in the past 10 years (Figure 2B). With time, the number of literatures published increased year by year. The period from 2012 to 2016 is a stage of slow growth, with an average of 93.4 literature published per year. The period from 2017 to 2021 is a stage of rapid growth, with an average of 229 kinds of literature published per year. The citations also increased with time, and the increase rate of citations increased significantly after 2018.

Literature in this field were published in 494 journals, and we summarized the top 10 journals (Table 2). The journal with the largest number of articles was the *Journal of Alzheimer’s Disease*, with a total of 68 articles, followed by *Molecular Neurobiology* (*n* = 59) and *International Journal of Molecular Sciences* (*n* = 50). The IF of all journals was over 3.000. Five journals had an IF of more than 5.000, and their Journal Citation Reports (JCR) area was in Q1. In addition, the journal with the highest SJR indicator was *Frontiers in Cellular Neuroscience* (1.661), followed by *Frontiers in Molecular Neuroscience* (1.581), and *Frontiers in Aging Neuroscience* (1.324).

**TABLE 2:** Top 10 journals with the most publications.

Rank	Journal	Publications	IF_2021_	JCR	SJR indicator _2021_	OA
1	*Journal of Alzheimer’s Disease*	68	4.160	NEUROSCIENCES Q2	1.225	Option
2	*Molecular Neurobiology*	59	5.682	NEUROSCIENCES Q1	1.271	Option
3	*International Journal of Molecular Sciences*	50	6.208	BIOCHEMISTRY and MOLECULAR BIOLOGY Q1; CHEMISTRY, MULTIDISCIPLINARY Q2	1.176	Yes
4	*Frontiers in Aging Neuroscience*	39	5.702	NEUROSCIENCES Q1; GERIATRICS and GERONTOLOGY Q2	1.324	Yes
5	*Scientific Reports*	29	4.379	MULTIDISCIPLINARY SCIENCES Q2	1.005	Yes
6	*Frontiers in Cellular Neuroscience*	28	6.147	NEUROSCIENCES Q1	1.661	Yes
7	*Frontiers in Neuroscience*	27	5.152	NEUROSCIENCES Q2	1.275	Yes
8	*Frontiers in Molecular Neuroscience*	25	6.261	NEUROSCIENCES Q1	1.581	Yes
9	*Aging-US*	23	5.955	CELL BIOLOGY Q2; GERIATRICS and GERONTOLOGY Q2	1.212	Yes
10	*Molecular Medicine Reports*	22	3.423	MEDICINE, RESEARCH and EXPERIMENTAL Q3; ONCOLOGY Q3	0.65	Option

IF, impact factor; JCR, Journal Citation Reports; SJR, SCImago Journal Rank; OA, open access.

### Countries/regions

**FIGURE 3:**
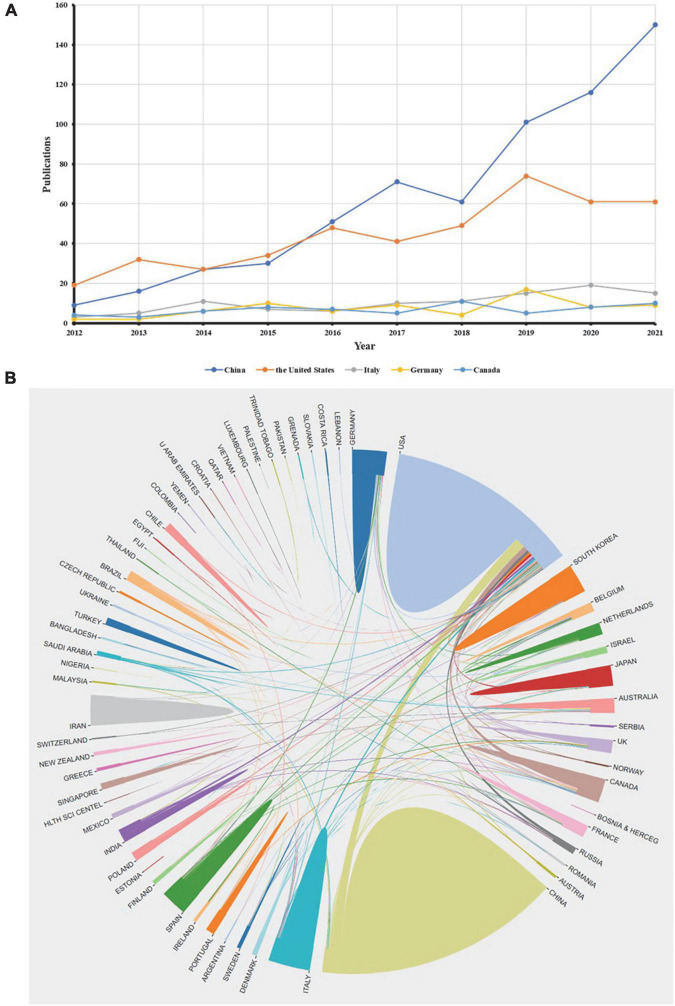
Countries/regions analysis in the field of AD and ncRNAs in the past decade. **(A)** The annual publications of the top five countries. **(B)** Cooperation among countries/regions in the field.

Researchers from 68 countries/regions published literature in this field. Among them, the country with the largest number of publications was China, with a total of 632 publications, followed by the United States (*n* = 446) and Italy (*n* = 102) (Table 3). From 2012 to 2015, the annual publications in the United States were very large. After 2016, the annual publications in China exceeded that of the United States, ranking first. The annual publications in Italy, Germany, and Canada were relatively small, but tended to be stable (Figure 3A). When we re-ranked the 10 countries according to the average citations, the top three countries were Canada (average citations = 45.42), Germany (average citations = 44.93), and the United States (average citations = 42.3). Moreover, although China had the largest number of publications, the number of countries/regions cooperating with China was fewer than that of the United States and Germany (Figure 3B). Particularly, the United States had the most cooperation in this field. In addition, Italy, Germany, and Canada, which rank third to fifth in terms of total publications, were also important nodes on the map of international cooperation.

**TABLE 3:** Top 10 countries/regions with the most publications.

Country/Region	Publications	Citations	Citations (without self-citations)	Average citations
China	632	16,349	14,528	25.87
The United States	446	18,866	17,640	42.3
Italy	102	3,213	3,141	31.5
Germany	73	3,280	3,223	44.93
Canada	67	3,043	2,979	45.42
India	60	1,695	1,685	28.25
South Korea	60	2,333	2,320	38.88
England	59	2,072	2,041	35.12
Spain	55	1,355	1,316	24.64
Iran	52	764	738	14.69

### Institutions

Researchers from a total of 1,843 institutions published literature in this field. We listed the top 10 institutions with the most published literature (Table 4). Louisiana State University System is the institution with the largest number of publications, with a total of 66 publications, followed by Louisiana State University Health Sciences Center New Orleans (*n* = 52) and the University of California System (*n* = 38). The institution with the highest average citation was Harvard University (average citations = 56.03) and followed by the University of California System (average citations = 48.5) and Louisiana State University Health Sciences Center New Orleans (average citations = 48.5).

**TABLE 4:** Top 10 institutions with the most publications.

Institution	Publications	Citations	Citations (without self-citations)	Average citations
Louisiana State University System	66	2,387	2,112	36.17
Louisiana State University Health Sciences Center New Orleans	52	2,134	1,901	41.04
University of California System	38	1,843	1,831	48.5
Shanghai Jiao Tong University	36	1,271	1,261	35.31
Central South University	35	852	840	24.34
Capital Medical University	33	793	777	24.03
Harvard University	32	1,793	1,772	56.03
Harbin Medical University	29	834	818	28.76
Huazhong University of Science and Technology	27	852	833	31.56
Texas Tech University	26	1,174	1,080	45.15

**FIGURE 4:**
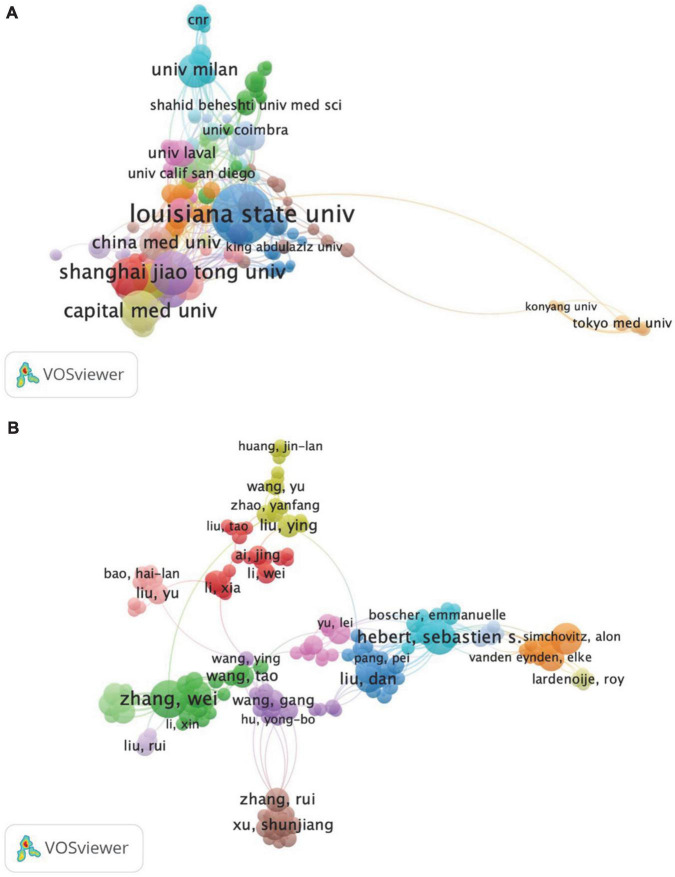
The map of institutions’ cooperation **(A)** and authors’ cooperation **(B)** in the field of AD and ncRNAs in the past decade.

The maps of institutions’ cooperation in the field of AD and ncRNAs are shown in Figure 4A, which consisted of 311 projects, 18 clusters, 950 links, and 1,321 total link strength. In terms of the number of links, Louisiana State University System had the largest number of links, with 58 lines, indicating the most cooperation with other organizations in this field, followed by Shanghai Jiao Tong University (link = 46) and Boston University (link = 34).

In addition, we found that Brigham and Women’s Hospital, Broad Institute, Diamir, LLC, Harvard University, and the University of Pennsylvania were associated with one cluster. Moreover, Biorchestra Co., Ltd., Griffith University, Konyang University, Meiji Pharmaceut University, Nagoya University, National Cancer Center Hospital, National Center for Geriatrics and Gerontology, RIKEN Center for Integrative Medical Sciences, Tokyo Medical University, University of South Pacific, and the University of Tokyo were related to another group with a long-distance.

### Authors

A total of 7,237 authors have published literature in this field, and the top three authors were Lukiw WJ (*n* = 56), Zhao YH (*n* = 37), and Wang Y (*n* = 29) (Table 5). The total citations of Lukiw WJ reached 2,212, and the average citation was 39.5. In addition, Lukiw WJ’s H-index reached 27, which was the author with the highest H-index in this field. Moreover, Reddy PH was the author with the highest average citations, up to 49.17, followed by Kumar S (average citations = 42.94) and Lukiw WJ (average citations = 39.5).

**TABLE 5:** Top 10 authors with the most publications.

Author	Affiliation*	Publications	Citations	Citations (without self-citations)	Average citations	H-index
Lukiw WJ	Louisiana State University	56	2,212	1,941	39.5	27
Zhao YH	Louisiana State University	37	1,388	1,305	37.51	22
Wang Y	Huazhong University of Science and Technology	29	672	664	23.17	17
Zhang Y	Qingdao University	25	917	902	36.68	16
Reddy PH	Texas Tech University	23	1,131	1,048	49.17	17
Liu Y	Inner Mongolia University Nationalities	20	357	357	17.85	9
Zhang L	Fudan University	19	410	409	21.58	10
Zhang W	Shanghai Jiao Tong University	19	686	678	36.11	12
Kumar S	Texas Tech University	18	773	719	42.94	13
Wang H	Shanghai Jiao Tong University	16	229	228	14.31	9

*The author’s affiliations were extracted from the search results for the most recently published article.

A visual analysis of the cooperation map between authors with three or more publications was performed using VOSviewer (Figure 4B). The principal researchers of the cooperation map were Lukiw WJ (Documents = 54, Citations = 2207, Total link strength = 117), Zhao YH (documents = 37, citations = 1390, total link strength = 108), Zhang W (documents = 19, citations = 689, total link strength = 76) and Hebert, SS (documents = 15, citations = 838, total link strength = 60). Other researchers were related to one of these lead researchers. Within the cooperation network, 14 major author clusters could be distinguished. Among them, the red cluster had the most collaborators, including Ai J, Liu Y, Yang Hui, Gao Y, Zhang Y, and Li W, followed by the green cluster and blue cluster.

### Keywords

**FIGURE 5:**
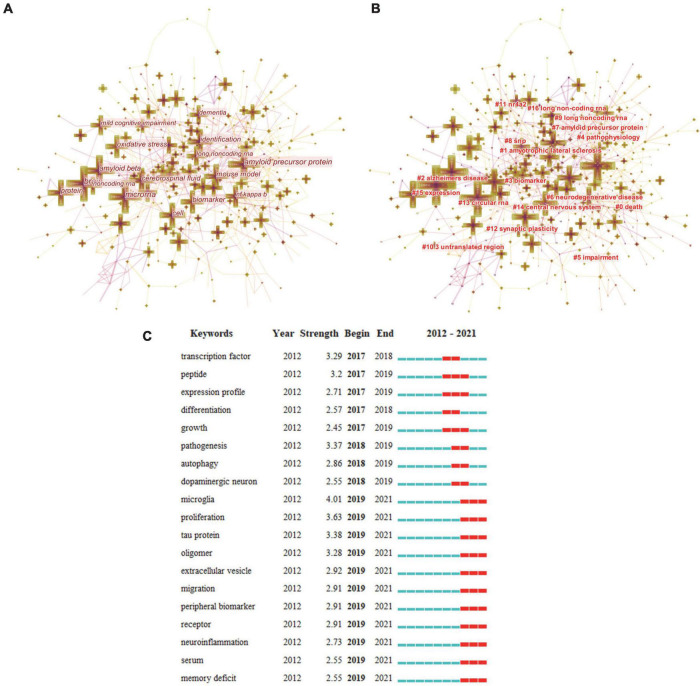
The map of keywords co-occurrence **(A)**, keywords clusters **(B)**, and burst keywords **(C)**.

CiteSpace was used to analyze keywords in the field of AD and ncRNAs. We deleted words such as expression, gene-expression and RNA that did not affect the subsequent analysis. CiteSpace was used to conduct a co-occurrence analysis of keywords (Figure 5A). A total of 494 nodes, 802 links were generated in the map of keywords co-occurrence, and we listed the top 10 co-occurrence keywords (Table 6). A total of 17 clusters were obtained by keyword cluster analysis (Figure 5B and Table 7), which had high credibility (the Silhouette value of each cluster was greater than 0.7). Besides, the burst analysis found that the burst words in the past 5 years (Figure 5C).

**TABLE 6:** Top 10 co-occurrence keywords *via* CiteSpace.

Keyword	Counts
Brain	258
MicroRNAs	216
Amyloid precursor protein	168
Biomarker	152
Amyloid beta	146
Cerebrospinal fluid	134
Protein	128
Identification	125
Mouse model	112
Oxidative stress	111

**TABLE 7:** The clusters in the field of AD and ncRNAs.

ID	Cluster	Size	Silhouette	Year
0	Death	50	0.865	2016
1	Amyotrophic lateral sclerosis	45	0.855	2015
2	Alzheimer’s disease	35	0.912	2014
3	Biomarker	32	0.862	2015
4	Pathophysiology	32	0.788	2017
5	Impairment	31	0.941	2017
6	Neurodegenerative disease	30	0.872	2016
7	Amyloid precursor protein	27	0.941	2014
8	SNP	25	0.854	2016
9	Long non-coding RNA	25	0.851	2014
10	3′ untranslated region	25	0.965	2014
11	Nr4a2	24	0.876	2017
12	Synaptic plasticity	23	0.936	2017
13	Circular RNA	23	0.915	2017
14	Central nervous system	23	0.861	2013
15	Expression	21	0.918	2015
16	Long non-coding RNA	20	0.86	2017

## Discussion

Non-coding RNAs play an important role in AD and are being explored by researchers in various countries. Through the methods of bibliometric and visual analysis, our study summarizes the research trends and hotspots, analyzes the cooperation in the field, and predicts the direction of future research in this field.

### High scientific research outputs and high-quality journals

In the field of AD and ncRNAs, review and article account for more than 94% of all document types, especially article accounts for more than 70%. The number of research outputs is increasing year by year, indicating that the field is developing well. Of note, from 2012 to 2016, the average annual number of publications in this field was fewer than 100. With the accumulation of relevant research basis, the average annual number of articles published from 2017 to 2021 has exceeded 200. Therefore, 2017 can be seen as an important inflection point for the rapid development of this field. This also indicates that since 2017, more and more studies have focused on ncRNAs. Moreover, the total citations of articles have increased sharply since 2018. This may be attributed to the gradual accumulation of previous studies, leading more and more researchers to pay attention to this field.

Journal analysis can provide effective references for the publication of research results. The *Journal of Alzheimer’s Disease* is a professional journal on AD and the most popular journal in this field, suggesting that researchers prefer to publish their findings in professional journals. In addition to the number of publications, publication quality is also one metric, and the SJR indicator can better help researchers find high-quality journals in their field (Falagas et al., 2008). In general, the SJR indicator has a certain positive correlation with journal impact factors. The more highly cited articles in the journal, the more likely they are to be cited by other prestige journals (Yuen, 2018). In the field of AD and ncRNAs, the top 10 journals with the most publications are almost high-quality journals. Among them, *Frontiers in Cellular Neuroscience*, *Frontiers in Molecular Neuroscience*, and *Frontiers in Aging Neuroscience* are good choices for the majority of researchers to publish relevant research results. Different from the previous bibliometric analysis, we summarize whether the journal is an Open Access (OA) journal (Benita, 2021; Li et al., 2021). OA journals can significantly increase the citations of literature because they provide a wider global readership than any subscription-based journal. In the top 10 journals, almost all journals offer OA options, which is also one of the trends in literature publication in recent years.

### Cooperation needs to be strengthened

Most of the previous studies show that the United States has the highest scientific productivity in many fields (Wang et al., 2020; Yao et al., 2020). Until 2016, the United States was ahead of any other country in annual publications, and since then, China has surpassed the United States in annual publications. However, China is the country that has the highest scientific productivity in this field. Unfortunately, China’s average citations are almost at the bottom among the top 10 countries/regions with the most publications, which means that China still lacks some influence in this field. Although the United States ranks second in scientific productivity, its literature has high average citations. Meanwhile, the United States has a lot of cooperation with other countries, which means that it is still the influential research country in this field.

In addition, the results of the institutions analysis show that most of the research in this field is still concentrated in scientific research institutions in the United States, which indirectly indicates that China’s institutions that publish scientific research outputs are relatively scattered and lack cooperation. Similar results can also be obtained through the Visualization analysis of VOSviewer. Few institutions have published high-cited research in China, and there is a lack of international cooperation. Besides, we further summarized the top 10 authors and analyzed the cooperation between the authors who published more than 3 articles. Among the authors with high scientific productivity, Chinese authors account for a large proportion. More importantly, Chinese authors with high productivity carry out more cooperation, but they still focus on domestic cooperation and lack of international cooperation.

Objects with high scientific productivity and influence in the field can be identified through the analysis of countries/regions, institutions, and authors. Through visualization analysis, the cooperation among them can be determined. Cooperation with producers (authors, institutions, countries/regions) with high publications is indeed an effective way for some producers where scientific research is in its infancy or early stages, and may help to increase the output and impact of scientific research. Additionally, the development of international cooperation is more conducive to the improvement of scientific research output, which needs to be strengthened in most countries, including China.

### The highly cited articles are instructive

Articles with high citations often have important reference value, and sometimes they may be key articles that can lay the foundation for a certain field (Cheng et al., 2022; Li Y. et al., 2022). The most cited article in this field is “Amyloid beta: structure, biology and structure-based therapeutic development” published by the research team of Chen et al. (2017). They review the role of Amyloid beta in AD. More importantly, they have mentioned that ncRNAs, especially microRNAs (miRNAs), have become a potential therapeutic target of AD, which boosts researchers’ confidence in exploring the relationship between AD and ncRNAs.

Besides, Lukiw WJ, the author with the highest scientific productivity, published “Deficiency in the Ubiquitin Conjugating Enzyme UBE2A in Alzheimer’s disease (AD) is Linked to Deficits in a Natural Circular miRNA-7 Sponge (circRNA; ciRS-7),” which is widely cited (Zhao et al., 2016). This article indicates that the occurrence of AD is related to the lack of ciRS-7. Of note, Reddy PH, the author with the highest average citations, published “Are circulating microRNAs peripheral biomarkers for Alzheimer’s disease?”, which is also worthy of attention (Kumar and Reddy, 2016). They reveal some promising candidates for miRNAs, such as miR-9, miR-125b, and let-7g-5p, and discuss their diagnostic features and cellular functions. These findings provide a basis for finding potential biomarkers of AD. From these highly cited articles, it can be found that ncRNAs play an important role in the diagnosis and treatment of AD and are expected to become a diagnostic biomarker and therapeutic target.

### Research hotspots and frontiers

#### Keywords co-occurrence analysis to preliminarily identify the hotspots in the field

The analysis of keywords co-occurrence can preliminarily identify the research hotspots in this field. The co-occurrence frequency of “brain,” “microRNAs,” “amyloid precursor protein,” “biomarker,” “amyloid beta,” “cerebrospinal fluid,” “protein,” “identification,” “mouse model,” and “oxidative stress” are all over 100. These results illustrate that the hotspots of research in this field are mainly focused on the role and mechanism of ncRNAs, especially miRNAs, in AD. And the level of research is mainly based on basic research, focusing on animal and cellular levels, and related to proteomics.

Indeed, such results are also in line with the current overall research trend in this field. It is well known that amyloid precursor protein and amyloid-beta are critical to the occurrence and development of AD. Anti-amyloid therapy is also a popular strategy in the treatment of AD in recent years (Brucki et al., 2022). More importantly, some ncRNAs can attenuate amyloid-induced neurotoxicity by targeting certain proteins. For example, miR-431 can protect amyloid-beta-induced nerve damage by inhibiting the expression of Kremen1 (Ross et al., 2018). Moreover, the latest study also found that inhibiting the expression of miR-26a-5p can up-regulate the expression of its target PTGS2, thereby alleviating amyloid-beta-induced nerve damage (Xie et al., 2022).

In addition, the two keywords “identification” and “cerebrospinal-fluid” have also attracted our attention. In recent years, most research on the role of ncRNAs from different sources in AD have been published. In fact, ncRNAs are not only found in nerve cells but can also be detected in blood and cerebrospinal fluid, suggesting that ncRNAs from different tissue may have different clinical significance (Nikolac Perkovic et al., 2021; Doroszkiewicz et al., 2022). In fact, the expression level of ncRNAs in different tissues may also be different, which also makes it difficult to explore biomarkers for AD. For example, compared with normal controls, the expression of hsa-miR-16-5p in peripheral blood samples is not significantly different, but significantly increases in cerebrospinal fluid (Asadi et al., 2022; Sandau et al., 2022).

#### Keywords cluster analysis to further identify the hotspots in the field

We used CiteSpace to divide these keywords into 17 clusters. The smaller the cluster number, the more keywords it contains. Through cluster analysis, the keywords can be better summarized and classified, and the research hotspots in this field can be better understood. At present, the research hotspots in this field are still focused on “death,” “amyotrophic lateral sclerosis,” “Alzheimer’s disease,” “biomarker,” “pathophysiology,” “impairment,” “neurodegenerative disease,” “amyloid precursor protein,” “single nucleotide polymorphisms (SNP),” “long non-coding RNA,” “3′ untranslated region,” “Nr4a2,” “synaptic plasticity,” “circular RNA,” and “central nervous system.”

“Alzheimer’s disease” and “amyotrophic lateral sclerosis” belong to neurodegenerative diseases of the central nervous system, and they have some similar pathogenesis (Almikhlafi et al., 2022; Li S. H. et al., 2022). Thus, it is not surprising that these labels can be formed after keyword clustering analysis. On the contrary, it is “death” that arouses our interest as the largest cluster in the field. We know that neurodegeneration is accompanied by nerve cell death, and anti-nerve cell death is one of the strategies for the treatment of AD (Liang and Wang, 2022). More importantly, ncRNAs can play a role in the regulation of nerve cell death, and ncRNAs are expected to become another therapeutic target for AD. It has been found that down-regulation of miR-22 can significantly alleviate the apoptosis of PC-12 cells induced by LPS and achieve the purpose of treating diseases (Wang Y. et al., 2022). Of course, there are many types of nerve cell death, including apoptosis, pyroptosis, necrosis, and ferroptosis (He et al., 2022; Jin et al., 2022; Mangalmurti and Lukens, 2022). Whether ncRNAs can achieve the purpose of treating diseases by regulating different types of death is also worthy of further study in the future (Li W. et al., 2022; Wang J. et al., 2022).

It is worth mentioning that the cluster “Nr4a2” has also attracted our attention. It has been reported that the transcriptional factor nuclear receptor 4A subfamily (Nr4a) is involved in a variety of physiological processes in the hippocampus, from inflammation to neuroprotection, and may become a new synaptic therapy target for AD (Català-Solsona et al., 2021). More importantly, ncRNAs may also regulate Nr4a to play a role in the treatment of AD (Annese et al., 2018; Zhang et al., 2021). For example, some studies have found that miR-409-3p activates the NF-κB pathway by targeting Nr4a2, which promotes neuroinflammation (Hu et al., 2021). Therefore, regulating the expression of ncRNAs can achieve the purpose of regulating related target proteins, which is also one of the hotspots concerned by researchers in this field.

#### Keywords burst analysis identify the frontiers in the field

We summarize the burst keywords in the past 5 years, in which the burst of “microglia,” “proliferation,” “tau protein,” “oligomer,” “extracellular vesicle,” “migration,” “peripheral biomarker,” “receptor,” “neuroinflammation,” “serum,” and “memory deficit” continued until 2021. These burst keywords may be trends for future research. For example, neuroinflammation, as a common phenotype in the pathological state of nerve cells, has been paid more and more attention. And revealing the regulatory mechanism of ncRNAs in AD from the perspective of neuroinflammation can provide new ideas for the treatment of AD (Lan et al., 2021; Zingale et al., 2021). A recent review article also shows that ncRNAs in exosomes can mediate nerve inflammation and promote the development of AD (Weng et al., 2022). In addition, memory deficits are the main characteristics of AD, and improving memory deficits by regulating ncRNAs is also a future research direction (Ju et al., 2021; Xiao et al., 2021; Zhang G. J. et al., 2022).

In short, through the keyword burst analysis, we can objectively predict the future research hotspots and guide researchers to choose the future research direction. The results of burst analysis are friendly to the emerging research team, and these different topics, such as the application of circular RNAs from different tissue sources in AD, the regulation of neuroinflammation, and tau protein, can be a good choice for these teams.

### Strengths and limitations

Previous bibliometric analysis related to AD have been published, such as intestinal flora and AD, epilepsy and AD, and brain energy metabolism disorder in AD (Du et al., 2021; Trejo-Castro et al., 2022; Zhang G. F. et al., 2022). These studies have revealed the research hotspots and frontiers in different subfields of AD. However, to the best of our knowledge, our study is the first bibliometric analysis in the field of AD and ncRNAs. The data involved in our study are all extracted from the Web of Science, and the extracted data are comprehensive, reliable, and highly recognized. A large number of previous bibliometric analysis have also used this database (Yao et al., 2020; Sun et al., 2022; You et al., 2022).

However, there are some limitations to our study. Our search strategy may not be able to retrieve all kinds of literature in this field, which is an inherent limitation of bibliometrics. As there is no restriction on the document types of literature, some authors may not publish research article, but mainly publish document type of review or letter, which may produce misleading results on contributions in this field, but this is unavoidable in bibliometric analysis (Zhu et al., 2021). Besides, the searched authors may have the same name or the change of their institutions, which may lead to bias in the results. In addition, as the year 2022 has not yet ended, it is impossible to accurately analyze the research hotspots of this year, so our bibliometrics does not include the literature published in 2022, but only discusses them. Therefore, we believe that the citations of the latest publications should be further analyzed in the future.

## Conclusion

Taken together, the field of AD and ncRNAs is developing well. *Journal of Alzheimer’s Disease* is a professional journal on AD and the most popular journal in this field. China is the country with the highest scientific productivity, but its scientific research influence needs to be improved. The United States has carried out the most international cooperation and remains the most influential country in the field. Louisiana State University System is the institution with the highest scientific productivity. Lukiw WJ is the most influential author. The research hotspots in this field focus on the role and mechanism of ncRNAs, especially miRNAs, in AD. The level of research in the field is mainly based on basic research, focusing on animal and cellular levels, and related to proteomics. Circular RNAs, regulation of neuroinflammation, and tau protein are the future research directions.

## Data availability statement

The original contributions presented in this study are included in the article/Supplementary material, further inquiries can be directed to the corresponding author/s.

## Author contributions

YG and YH contributed to the study conception and design and revised the manuscript. XF, SW, and JL performed the literature search and data analysis. XF, SW, QZ, and YG wrote the first draft of the manuscript. All authors listed have made a substantial contribution to the work, read, and approved the final manuscript.

## Footnotes

1
https://bibliometric.com/

## Abbreviations

AD: Alzheimer’s disease
circRNAs: circular RNAs
IF: impact factor
JCR: Journal Citation Reports
lncRNAs: long non-coding RNAs
miRNAs: microRNAs
ncRNAs: non-coding RNAs
NEAT1: nuclear paraspeckle assembly transcript1
Nr4a: nuclear receptor 4A subfamily
SJR: SCImago Journal Rank
SNP: single nucleotide polymorphisms.
