# Supplementary material for: Bibliometric analysis of research on Alzheimer’s disease and non-coding RNAs: Opportunities and challenges
Source: Front Aging Neurosci. 2022 Oct 18;14:1037068. doi: 10.3389/fnagi.2022.1037068 (PMC9623309; doi:10.3389/fnagi.2022.1037068)
Supplement: Supplementary file 2 [file Data_Sheet_2.PDF]

The parameter Settings of each visualization tool

VOSviewer

Specific parameters were set according to previous studies as follows: “Choose type of data” selected “Create a map based on bibliographic data”; “Choose data source” selected “Read data from bibliographic database files”; “Type of analysis” selected “Co-authorship”; “Unit of analysis” selected “Authors” or “Organizations”; “Counting method” selected “Full counting”; "Choose thresholds" option was adjusted according to the data, and the remaining Options were default.

CiteSpace

Specific parameters were set according to previous studies as follows: “Time Slicing” was set to 2012–2021 (#Years Per Slice = 1); “Term Source” selected “Title,” “Abstract,” “Author Keywords (DE),” and “Keywords Plus (ID)”; “Node Types” selected to “Keywords”; “Links” and “Selection Criteria” used the default option; “Pruning” selected “pathfinder” and “pruning the merged network”; “Visualization” selected “cluster view-static” and “show merged network.”

Bibliometric Visualization Website

After searching all the literature according to the search strategy, the results were exported to the "Win, UTF-8" format file. Open web analytics website (https://bibliometric.com/app). Click the button to upload data, and then select the corresponding analysis function on the left side of the web page to obtain the analysis results on the right side of the web page.
